# Pristane induced lupus mice as a model for neuropsychiatric lupus (NPSLE)

**DOI:** 10.1186/s12993-023-00205-y

**Published:** 2023-02-10

**Authors:** Yang Yun, Xuejiao Wang, Jingyi Xu, Chenye Jin, Jingyu Chen, Xueru Wang, Jianing Wang, Ling Qin, Pingting Yang

**Affiliations:** 1 Department of Nephrology, Shengjing Hospital of China Medical University, Shenyang, China; 2 Department of Physiology, China Medical University, Shenyang, China; 3 Department of Rheumatology and Immunology, First Affiliated Hospital, China Medical University, Shenyang, China

**Keywords:** Neuropsychiatric lupus, Mouse model, Behavioral deficit, Cytokine, IgG, Glia cells, Lipofuscin

## Abstract

**Background:**

The pristane-induced lupus (PIL) model is a useful tool for studying environmental-related systemic lupus erythematosus (SLE). However, neuropsychiatric manifestations in this model have not been investigated in detail. Because neuropsychiatric lupus (NPSLE) is an important complication of SLE, we investigated the neuropsychiatric symptoms in the PIL mouse model to evaluate its suitability for NPSLE studies.

**Results:**

PIL mice showed olfactory dysfunction accompanied by an anxiety- and depression-like phenotype at month 2 or 4 after pristane injection. The levels of cytokines (IL-1β, IFN-α, IFN-β, IL-10, IFN-γ, IL-6, TNF-α and IL-17A) and chemokines (CCL2 and CXCL10) in the brain and blood–brain barrier (BBB) permeability increased significantly from week 2 or month 1, and persisted throughout the observed course of the disease. Notably, IgG deposition in the choroid plexus and lateral ventricle wall were observed at month 1 and both astrocytes and microglia were activated. Persistent activation of astrocytes was detected throughout the observed course of the disease, while microglial activation diminished dramatically at month 4. Lipofuscin deposition, a sign of neuronal damage, was detected in cortical and hippocampal neurons from month 4 to 8.

**Conclusion:**

PIL mice exhibit a series of characteristic behavioral deficits and pathological changes in the brain, and therefore might be suitable for investigating disease pathogenesis and for evaluating potential therapeutic targets for environmental-related NPSLE.

**Supplementary Information:**

The online version contains supplementary material available at 10.1186/s12993-023-00205-y.

## Background

Systemic lupus erythematosus (SLE) is an intractable, multisystemic and relapsing disease characterized primarily by the loss of tolerance to self-antigens, immune complex formation and diverse end-organ damages [1]. Nervous system involvement in SLE affecting cognition, mood and the level of consciousness is termed neuropsychiatric lupus (NPSLE), and is associated with a worse prognosis and more cumulative organ damage [2, 3]. As for the pathogenesis of NPSLE, it is generally believed that several pathogenic factors, such as autoantibodies, immune cells and inflammatory mediators, may disrupt the blood–brain barrier (BBB) and promote an inflammatory process causing glial activation, neurodegeneration and behavioral deficits [4–6]. However, the mechanisms underlying NPSLE remain largely unknown.

Human research on NPSLE is limited by the difficulty of brain biopsy. Although histological analysis can be conducted on autopsy, rapid decomposition of brain tissue severely impacts the findings. Therefore, animal models of NPSLE are indispensable tools for exploring the pathogenic mechanisms [7]. MRL/lpr and NZB/W F1 mice are commonly used as NPSLE animal models. These mice spontaneously develop neuropsychiatric symptoms such as cognitive dysfunction [8, 9], anxiety and/or depression-like behavior [8, 10, 11] and decreased locomotor activity [12]. However, these models rely on inbred mouse strains, exhibit delayed and inconsistent onset of SLE-related symptoms and progress slowly [13]. The heterogeneous and mild neuropsychiatric symptoms limit the application of these models for NPSLE research. Additionally, the impact of environmental factors in the pathogenesis of NPSLE can not be fully reflected by these genetically predisposed models.

**Fig. 1:**
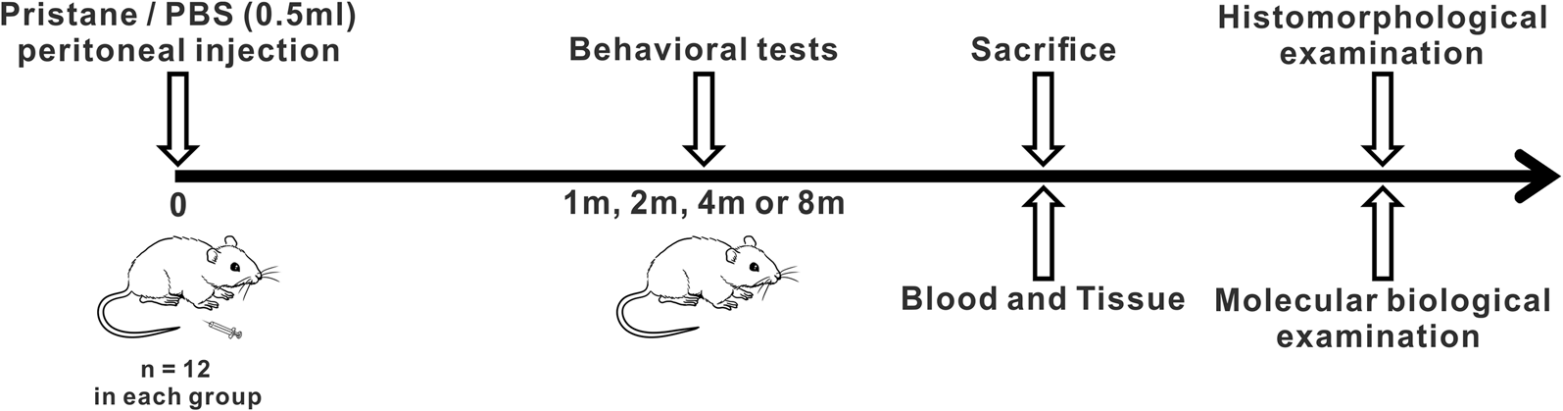
Experimental schedule. Mice were administered pristane (0.5 ml) or PBS via intraperitoneal injection. After a battery of behavioral tests, mice were sacrificed at month 1, 2, 4 or 8. Blood and tissue samples were collected for further histomorphological and molecular biological assays

Exposing wild-type mice to chemical agents is an alternative method for establishing SLE models, and can mimic the impact of environmental factors on the induction of lupus-specific manifestations. Such models allow investigation of non-genetic factors initiating a breakdown of immune tolerance and can be used to evaluate the efficacy of SLE therapeutic drugs. Pristane is known as hydrocarbon oil (2,6,10,14-tetramethylpentadecane) that can induce a wide range of autoantibodies specific to or associated with SLE in mice. In nature, this oil can be found in small amounts in vegetables [14], in the liver of some sharks [15], and as a byproduct of petroleum distillation [14]. Administration of pristane into the abdominal cavity of mice can cause a series of lupus-like manifestations, including ascitic fluid enriched with monoclonal antibodies, lipogranulomas, rheumatoid-like erosive arthritis and diffuse proliferative glomerulonephritis [16]. Though most of the major clinical and laboratory manifestations of SLE well described by the American College of Rheumatology are observed in this pristane-induced lupus (PIL) model, there are few reports on NPSLE manifestations in this model [17–19]. For this, we combined behavioral, histomorphological and biochemical approaches to comprehensively evaluate brain dysfunctions and pathological changes in the PIL model (Fig. 1). Our findings highlight the significance of the PIL model for research on the cellular and molecular mechanisms underlying environmental-related NPSLE.

## Results

### Lupus-like manifestations in PIL mice

**Fig. 2:**
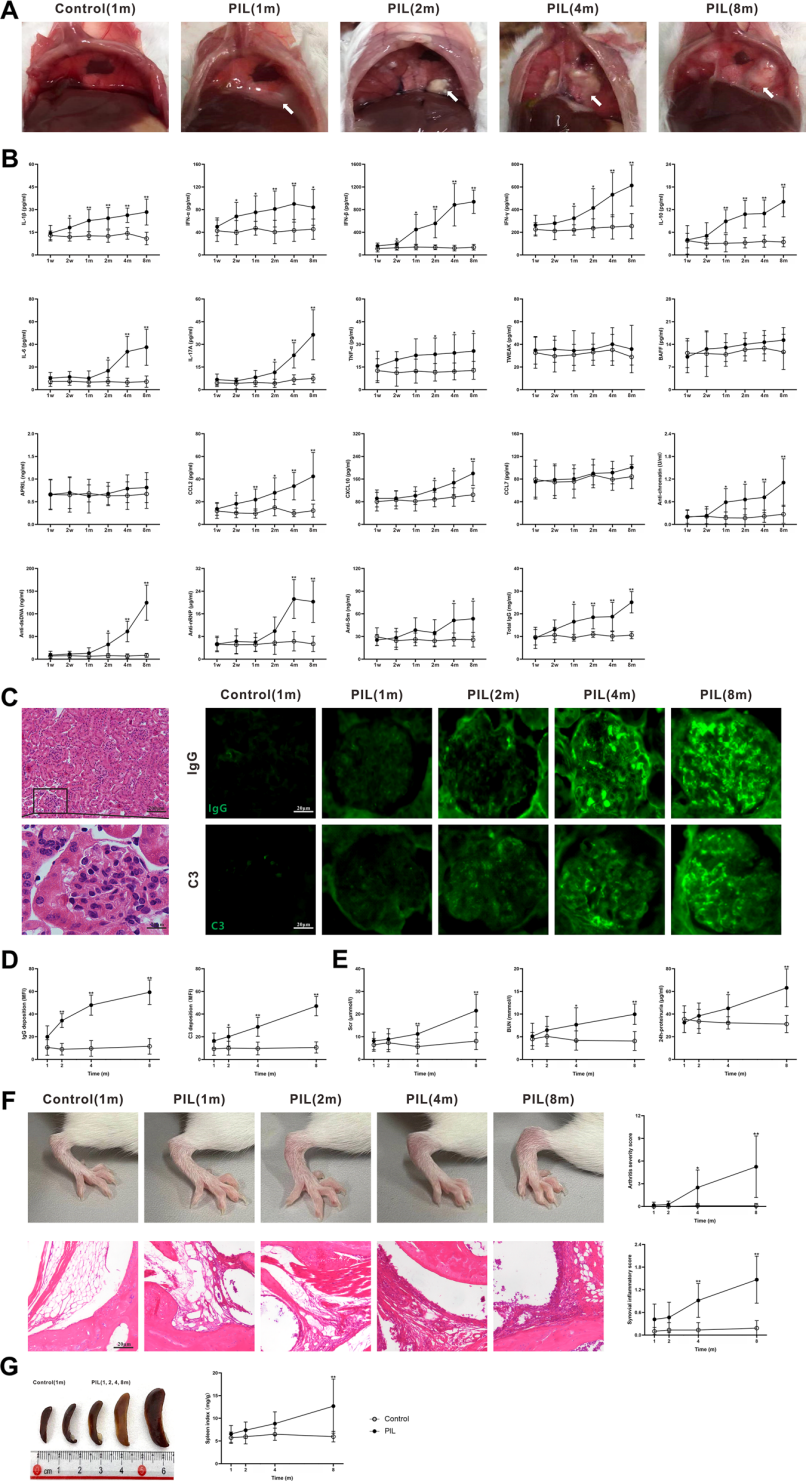
Examination for lupus-like symptoms in PIL mice. **A** Representative images showing lipogranulomas (arrow) adherent to the abdominal surface of the diaphragm in PIL mice. **B** Serum levels of cytokines (IL-1β, IFN-α, IFN-β, IFN-γ, IL-10, IL-6, IL-17A, TNF-α, TWEAK, BAFF and APRIL), chemokines (CCL2, CXCL10 and CCL7), autoantibodies (anti-chromatin, anti-dsDNA, anti-nRNP and anti-Sm) and total IgG, as detected by ELISA. IL-1β (group effect: F_1,22_ = 144.5, *p* < 0.0001; time effect: F_3.991,87.81_ = 5.593, *p* = 0.0005; interaction effect: F_5,110_ = 6.255,* p* < 0.0001); IFN-α (*p* < 0.0001, 0.0472, 0.0754); IFN-β (*p* < 0.0001, < 0.0001, < 0.0001); IFN-γ (*p* < 0.0001, < 0.0001, < 0.0001); IL-10 (*p* < 0.0001, < 0.0001, < 0.0001); IL-6 (*p* < 0.0001, < 0.0001, < 0.0001); IL-17A (*p* < 0.0001, < 0.0001, < 0.0001); TNF-α (*p* < 0.0001, 0.3646, 0.3835); TWEAK (*p* = 0.0657, 0.7505, 0.9879); BAFF (*p* = 0.0538, 0.1978, 0.6215); APRIL (*p* = 0.2328, 0.8839, 0.8463); CCL2 (*p* < 0.0001, 0.0004, < 0.0001); CXCL10 (*p* < 0.0001, < 0.0001, 0.0037); CCL7 (*p* = 0.0985, 0.1661, 0.7891); anti-chromatin (*p* < 0.0001, 0.0004, < 0.0001); anti-dsDNA (*p* < 0.0001, < 0.0001, < 0.0001); anti-nRNP (*p* < 0.0001, < 0.0001, < 0.0001); anti-Sm (*p* < 0.0001, 0.0091, 0.0012) and total IgG (*p* < 0.0001, < 0.0001, < 0.0001). **C** Left panel showing representative images of glomeruli stained with H&E. Right panel showing representative images of frozen kidney sections stained for IgG and C3. **D** Quantitative analysis of the MFI of IgG (*p* < 0.0001, < 0.0001, < 0.0001) and C3 (*p* < 0.0001, < 0.0001, < 0.0001) in glomeruli. **E** Quantitative analysis of Scr (*p* < 0.0001, < 0.0001, < 0.0001), BUN (*p* < 0.0001, 0.0430, 0.0024) and 24 h proteinuria (*p* < 0.0001, 0.0001, < 0.0001). **F** Upper panel showing representative images of the hind paw, and quantitative analysis of arthritis severity score (*p* < 0.0001, 0.0004, < 0.0001). Lower panel showing representative images of joint tissues with H&E staining, and quantitative analysis of synovial inflammatory score (*p* < 0.0001, < 0.0001, < 0.0001). **G** Representative images of spleen, and quantitative analysis of spleen index (*p* = 0.0007, 0.0013, 0.0001). The data are expressed as the mean ± SEM (*n* = 12 in each group). Two-way ANOVA followed by *Tukey’s *post hoc test or *Scheirer–Ray–Hare* test: *p < 0.05, **p < 0.01

Consistent with previous studies [18, 20, 21], PIL mice exhibited several representative lupus-like manifestations, including lipogranuloma, elevated serum cytokines, chemokines and autoantibodies, immune complex-mediated nephritis with renal failure and proteinuria, arthritis, as well as splenomegaly (Fig. 2). Lipogranuloma adherent to the abdominal surface of the diaphragm appeared at month 1 after pristane injection, and progressed over time (Fig. 2A). Meanwhile, the serum levels of the cytokines interleukin-1β (IL-1β), interferon-α (IFN-α), IFN-β, IFN-γ, IL-10, IL-6, IL-17A and tumor necrosis factor-α (TNF-α) were elevated in PIL mice compared with their age-matched controls, from week 2, or month 1 or 2 (Fig. 2B). However, no significant difference between PIL and control mice was observed in the levels of tumor necrosis factor-like weak inducer of apoptosis (TWEAK), B cell activating factor (BAFF) or a proliferation-inducing ligand (APRIL) (Fig. 2B). Compared with control mice, the serum levels of chemokines C–C motif chemokine 2 (CCL2) and C-X-C motif chemokine 10 (CXCL10) were significantly elevated in PIL mice at week 2 or month 2, whereas no significant difference was found in serum CCL7 level (Fig. 2B). Similar to most cytokines, the levels of serum autoantibodies (anti-chromatin, anti-double stranded DNA (anti-dsDNA), anti-nuclear ribonucleoprotein (anti-nRNP) and anti-Sm) and serum total immunoglobin G (IgG) were significantly increased in PIL mice from month 1, 2 or 4 (Fig. 2B). Granular deposits of IgG and complement 3 (C3) were observed in the glomeruli of PIL mice (Fig. 2C). The mean fluorescent intensity (MFI) of IgG and C3 increased significantly at month 2, and further increased with time (Fig. 2D). As important indexes of renal injury, the levels of serum creatinine (Scr) and blood urea nitrogen (BUN) were detected to assess renal function. Compared with control mice, the levels of Scr and BUN were markedly elevated n PIL mice at month 4 and further increased as the disease progressed (Fig. 2E). Throughout the observation period, the average 24 h urine protein level was higher in PIL mice compared with control mice from month 4, and the difference was statistically significant (Fig. 2E). At month 4, symmetrical swelling appeared in the hind paws of PIL mice (Fig. 2F). Compared with control mice, the arthritis severity score showed a significant elevation in PIL mice from month 4 to 8 (Fig. 2F). In PIL mice, histologic sections of joint tissue showed the typical sign of severe arthritis with increased inflammatory cell infiltration in the synovial sub-lining (Fig. 2F). Similar to the arthritis severity score, the synovial inflammatory score in PIL mice was significantly increased at month 4, and gradually increased further as the disease progressed (Fig. 2F). Splenomegaly was observed in PIL mice, and the increase in the spleen index became significant at month 8 (Fig. 2G). Taken together, these results suggest that PIL mice exhibit typical lupus-like manifestations during the observed course of the disease.

### Olfactory dysfunction and an anxiety- and depression-like phenotype in PIL mice

**Fig. 3:**
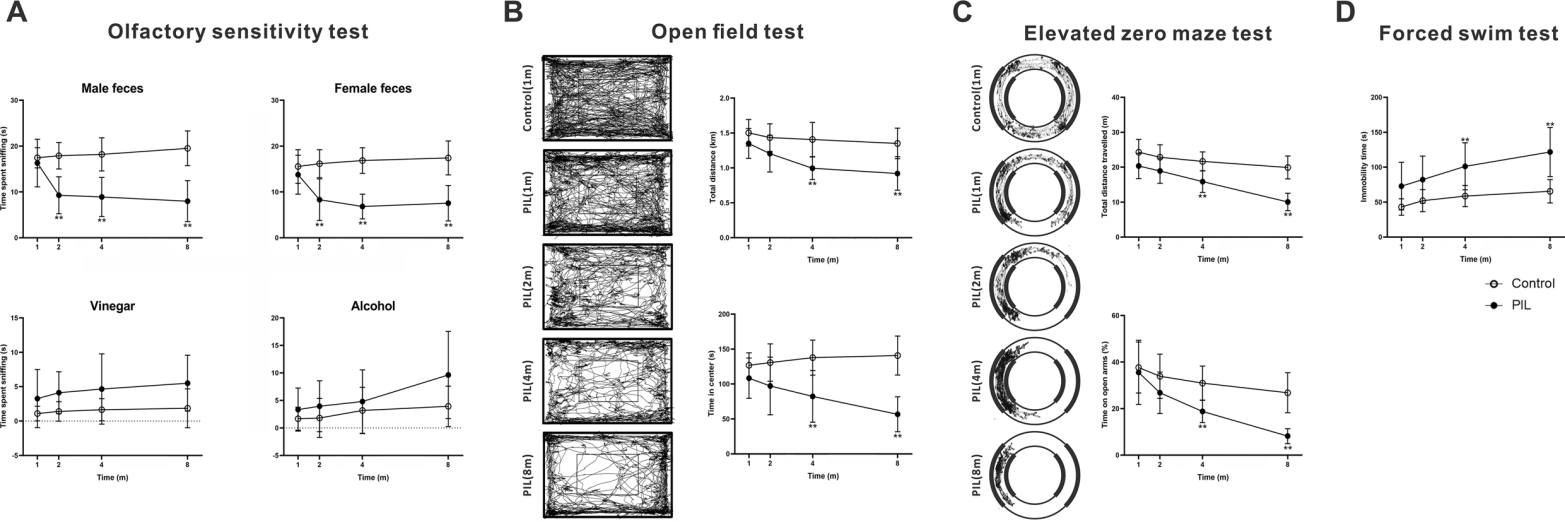
Behavioral assessments of olfactory function and anxiety- and depression-like phenotype. **A** Olfactory sensitivity test. Time spent sniffing male feces (*p* < 0.0001, 0.0037, < 0.0001), female feces (*p* < 0.0001, 0.0378, 0.0003), vinegar (*p* = 0.0035, 0.2964, 0.8263) or alcohol (*p* = 0.0055, 0.0248, 0.4079). **B** Open field test. Left panel showing representative images of the travelled path. Right panel showing quantitative analysis of the total distance travelled (*p* < 0.0001, 0.0001, 0.0925) and time spent in the center (*p* < 0.0001, 0.1251, 0.0007). **C** Elevated zero maze test. Left panel showing representative images of the travelled path. Right panel showing quantitative analysis of the total distance travelled (*p* < 0.0001, < 0.0001, 0.0056) and time spent in the open arms (%) (*p* < 0.0001, < 0.0001, 0.0201). **D** Forced swim test. Quantitative analysis of immobility time (*p* < 0.0001, 0.0009, 0.3390). The data are expressed as the mean ± SEM (*n* = 12 in each group). Two-way ANOVA followed by *Tukey’s *post hoc test or *Scheirer–Ray–Hare* test: **p < 0.01

**Fig. 4:**
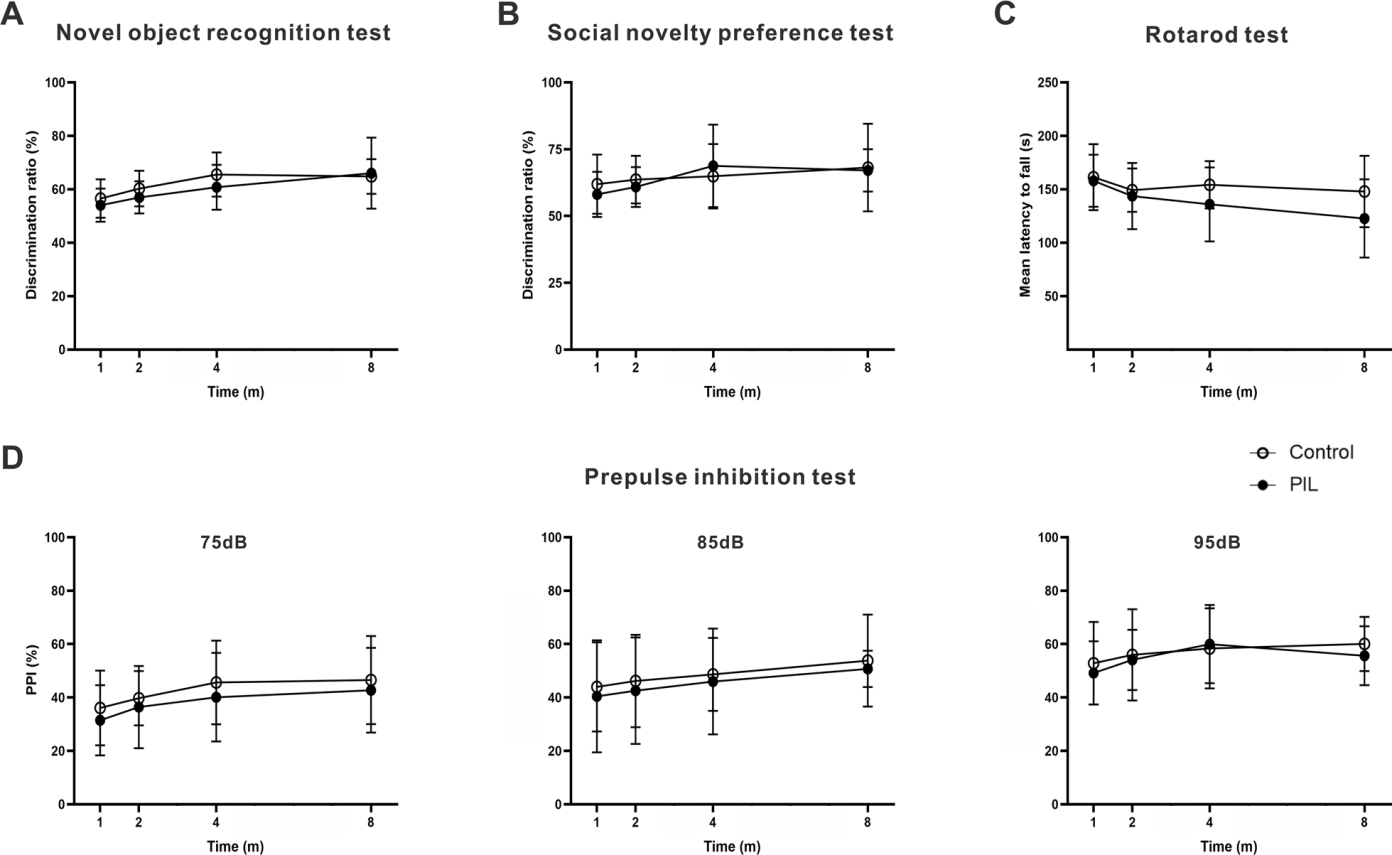
Behavioral assessments of novel object recognition, social novelty preference, rotarod and PPI tests. **A** Novel object recognition test. Quantitative analysis of the discrimination ratio (*p* = 0.1635, 0.0004, 0.6247). **B** Social novelty preference test. Quantitative analysis of the discrimination ratio (*p* = 0.6642, 0.0987, 0.6737). **C** Rotarod test. Quantitative analysis of the mean latency to fall (*p* = 0.0625, 0.0534, 0.5005). **D** Quantitative analysis of PPI% at 75 dB (*p* = 0.1491, 0.0777, 0.9946), 85 dB (*p* = 0.4376, 0.1693, 0.9995) or 95 dB (*p* = 0.4960, 0.1589, 0.8579). The data are expressed as the mean ± SEM (*n* = 12 in each group). Two-way ANOVA followed by *Tukey’s *post hoc test or *Scheirer–Ray–Hare* test

We used a battery of behavioral tests to assess the neuropsychiatric phenotype of PIL mice. As shown in Fig. 3A, PIL mice spent less time sniffing male or female fecal dilute solution than their age-matched controls from month 2. Although not statistically significant at any testing time point, an increase in the time spent sniffing vinegar and alcohol was also observed in PIL mice (Fig. 3A). These results indicate that PIL mice show reduced interest toward attractants, especially biological odorants, as well as decreased sensitivity to repellants. In the open field test, both the total tracked distance and the time spent in the center decreased over the course of the disease in PIL mice, compared with control mice, and the difference became significant at month 4 (Fig. 3B). Consistent with the results in the open field test, PIL mice exhibited anxiety-like behavior in the elevated zero maze test, as evidenced by significantly decreased total tracked distance and time spent in the open arms from month 4, and further decreased with time (Fig. 3C). Furthermore, immobility time in the forced swim test was significantly increased in PIL mice from month 4 to 8 (Fig. 3D). There were no differences between PIL mice and their age-matched controls in the novel object recognition test, social novelty preference test, rotarod test or prepulse inhibition (PPI) test at any testing time point (Fig. 4). Therefore, these findings suggest that deficits in olfactory function precede the anxiety- and depression-like phenotype in PIL mice.

### Overproduction of cytokines and chemokines in the brain and BBB leakage in PIL mice

**Fig. 5:**
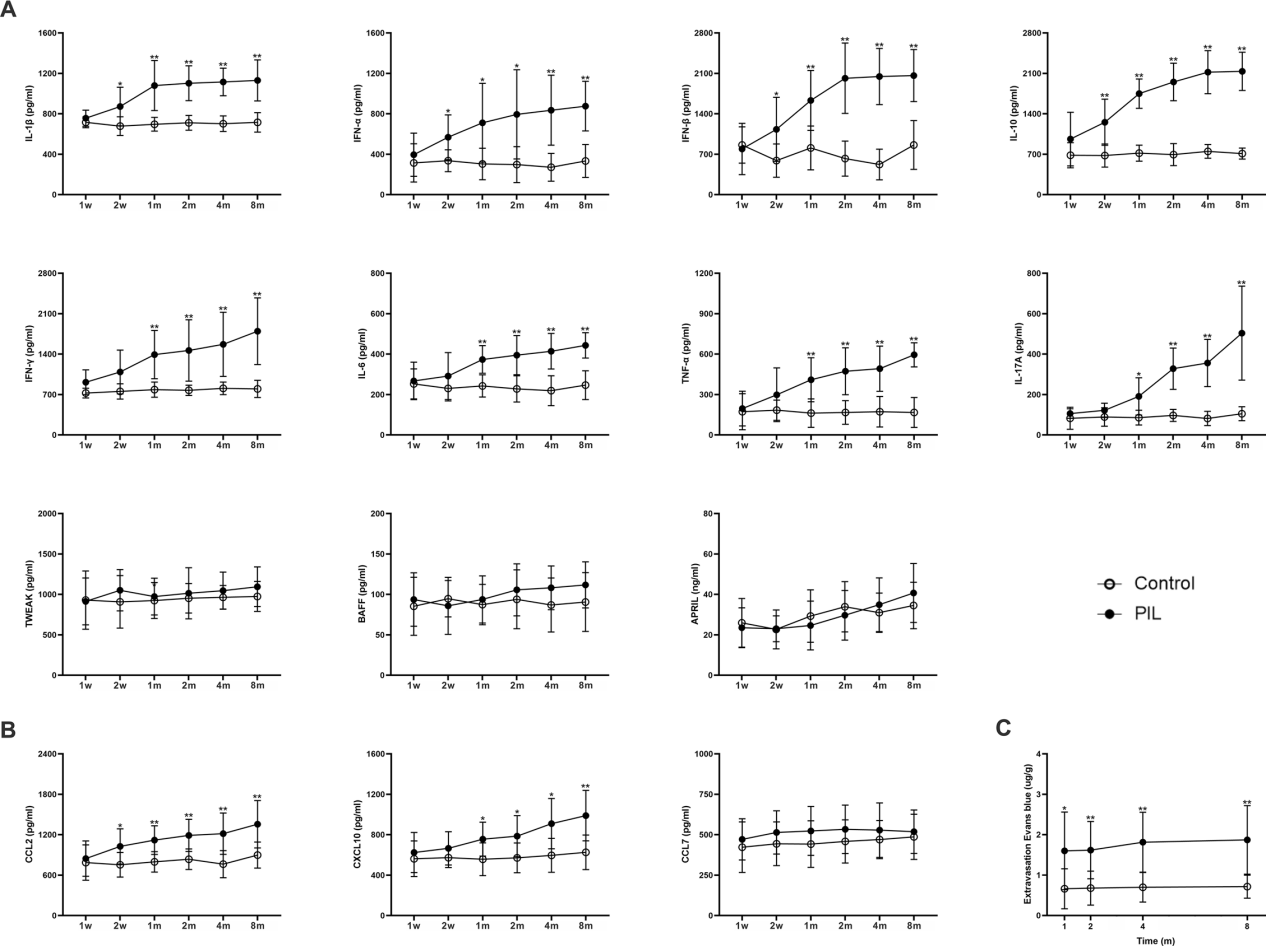
Levels of brain cytokines and chemokines, and BBB permeability. **A** Brain levels of cytokines (IL-1β, IFN-α, IFN-β, IL-10, IFN-γ, IL-6, TNF-α, IL-17A, TWEAK, BAFF and APRIL), as detected by ELISA. IL-1β (*p* < 0.0001, < 0.0001, < 0.0001); IFN-α (*p* < 0.0001, 0.0149, 0.0016); IFN-β (*p* < 0.0001, < 0.0001, < 0.0001); IL-10 (*p* < 0.0001, < 0.0001, < 0.0001); IFN-γ (*p* < 0.0001, 0.0002, 0.001); IL-6 (*p* < 0.0001, 0.0012, < 0.0001); TNF-α (*p* < 0.0001, < 0.0001, < 0.0001); IL-17A (*p* < 0.0001, < 0.0001, < 0.0001); TWEAK (*p* = 0.0562, 0.6937, 0.9245); BAFF (*p* = 0.0594, 0.6219, 0.5961) and APRIL (*p* = 0.9377, 0.0004, 0.4845). **B** Brain levels of chemokines (CCL2, CXCL10 and CCL7), as detected by ELISA. CCL2 (*p* < 0.0001, 0.0013, 0.0458); CXCL10 (*p* < 0.0001, 0.0016, 0.0295) and CCL7 (*p* = 0.0533, 0.6921, 0.9885). **C** Quantitative analysis of Evans blue dye extravasation (*p* < 0.0001, 0.6859, 0.8935). The data are expressed as the mean ± SEM (*n* = 12 in each group). Two-way ANOVA followed by *Tukey’s *post hoc test or *Scheirer–Ray–Hare* test: *p < 0.05, **p < 0.01

ELISA assays revealed that the levels of the cytokines IL-1β, IFN-α, IFN-β, IL-10, IFN-γ, IL-6, TNF-α and IL-17A and the chemokines CCL2 and CXCL10 were significantly elevated in the brain tissues of PIL mice compared with their age-matched controls at week 2 or month 1, and these high levels were maintained throughout the observed course of the disease (Fig. 5A, B). We found no significant difference in the levels of the cytokines TWEAK, BAFF and APRIL or the chemokine CCL7 between PIL and control mice (Fig. 5A, B). No obvious pathological changes, such as parenchymal lesions, ischemic lesions by vasculitis or thromboembolism or brain atrophy, were observed in the brain of PIL mice (data not shown). However, BBB permeability, determined by evaluating the extravasation of Evans blue, showed some abnormalities. Compared with age-matched controls, there was a marked increase in Evans blue content in the brain of PIL mice at month 1, and was maintained at a relatively stable level over the observed course of the disease (Fig. 5C).

### IgG deposition in the brain of PIL mice

**Fig. 6:**
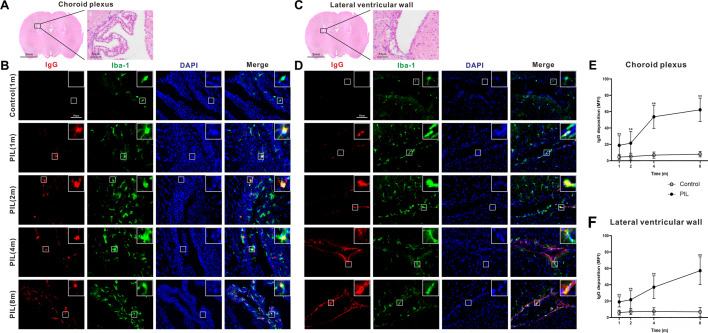
Examination for IgG deposition in the choroid plexus and lateral ventricular wall. **A**, **C** Representative images of the choroid plexus and lateral ventricular wall stained with H&E. **B**, **D** Representative images of IgG deposition in the choroid plexus and lateral ventricular wall. White square showing the co-localization of an enlarged Iba-1-immunoreactive microglia (green) and IgG (red). DAPI staining for nuclei (blue). **E**, **F** Quantitative analysis of IgG deposition in the choroid plexus (*p* < 0.0001, < 0.0001, < 0.0001) and lateral ventricular wall (*p* < 0.0001, < 0.0001, < 0.0001) by MFI. The data are expressed as the mean ± SEM (*n* = 12 in each group). Two-way ANOVA followed by *Tukey’s *post hoc test or *Scheirer–Ray–Hare* test: **p < 0.01

Immunofluorescence staining of brain sections revealed IgG deposition in the choroid plexus and lateral ventricular wall of PIL mice at month 1, and the deposits gradually increased as the disease progressed (Fig. 6). Some IgGs co-localized with microglia (stained for ionized calcium binding adapter molecule 1 (Iba-1)) in PIL mice (Fig. 6B, D). Because there was no difference in IgG deposition between the different testing time points in the age-matched controls, we show a representative image for the control group (at month 1) in Fig. 6B, D for brevity.

### Dynamic changes in microglia and astrocytes in the hippocampus of PIL mice

**Fig. 7:**
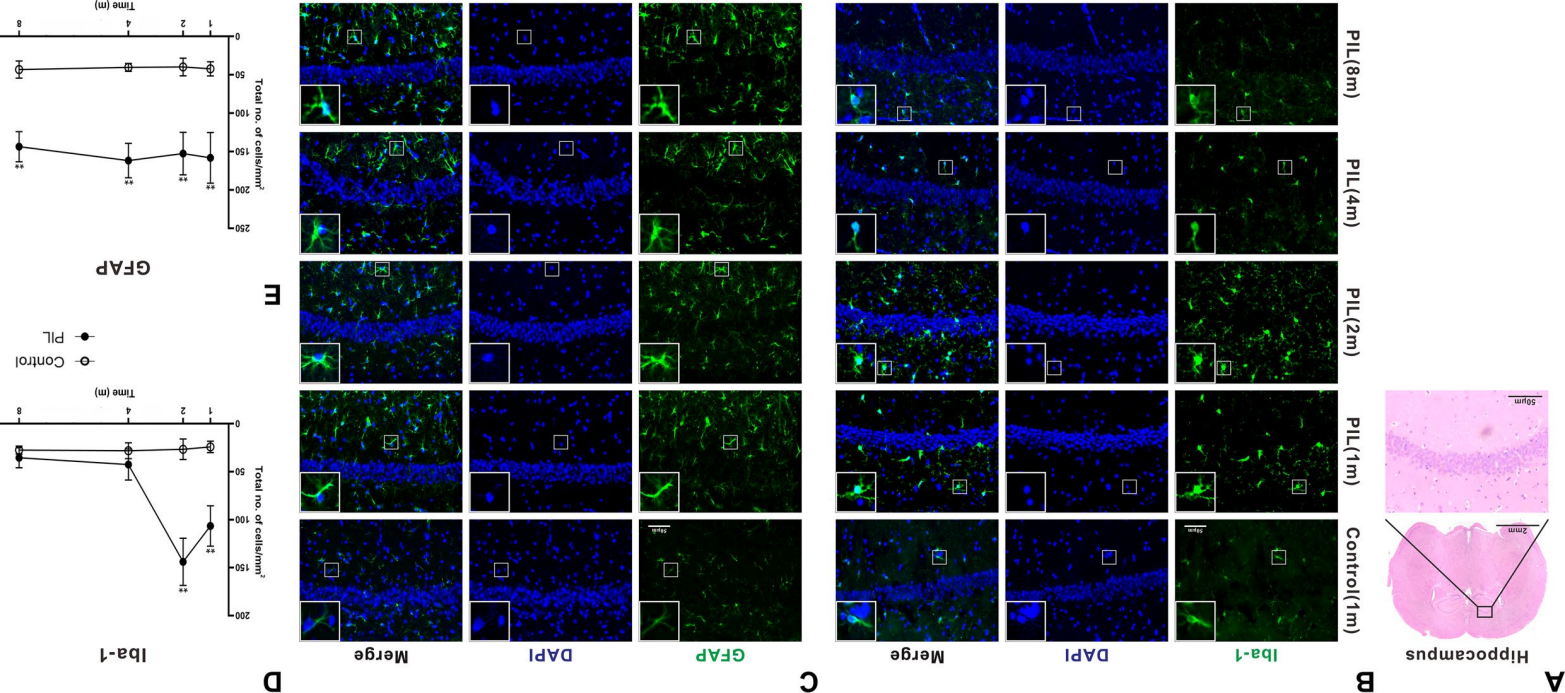
Density and morphology of microglia and astrocytes in the hippocampus. **A** Representative images of the hippocampus stained with H&E. **B**, **C** Representative images showing Iba-1-immunoreactive microglia and GFAP-immunoreactive astrocytes in the hippocampus. DAPI staining of nuclei (blue). White square showing an enlarged cell to compare morphology. **D**, **E** Quantitative analysis of the density of Iba-1-immunoreactive cells (*p* < 0.0001, < 0.0001, < 0.0001) and GFAP-immunoreactive cells (*p* < 0.0001, 0.5512, 0.3670). The data are expressed as the mean ± SEM (*n* = 12 in each group). Two-way ANOVA followed by *Tukey’s *post hoc test or *Scheirer–Ray–Hare* test: **p < 0.01

Because the hippocampus plays a critical role in olfactory function [22] and mood regulation [23], our behavioral tests suggest an involvement of the hippocampus in PIL mice. We therefore conducted immunohistochemical staining on the brain slices to examine the effects of pristane injection on glial cells in the hippocampus. We compared the distribution of cells immunoreactive for the microglial marker Iba-1. Compared with control mice, the density of Iba-1-immunoreactive microglia was increased significantly in PIL mice at month 1, reached a peak at month 2, and then declined sharply (Fig. 7B, D). Notably, the microglia in PIL mice showed a phenotypic transformation from the ramified to an activated state characterized by short retracted processes and a large irregular cell soma (Fig. 7B). Immunofluorescence staining revealed that the density of glial fibrillary acid protein (GFAP)-immunoreactive astrocytes was significantly increased in the hippocampus of PIL mice at month 1 and maintained throughout the observed disease course (Fig. 7C, E), accompanied by a change to an activated morphology (Fig. 7C). These results suggest that the behavioral deficits in PIL mice are accompanied by dynamic changes in microglia and astrocytes.

### Lipofuscin deposition in the brain of PIL mice

**Fig. 8:**
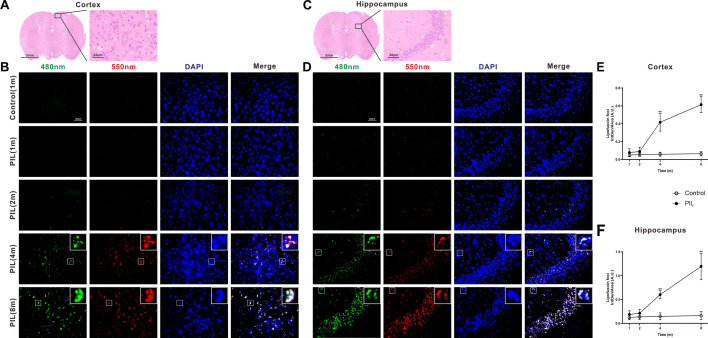
Examination for lipofuscin deposition in the cortex and hippocampus. **A** and **C** Representative images of the cortex and hippocampus stained with H&E. **B** and **D** Representative images showing autofluorescent lipofuscin at 480 nm (green) and 550 nm (red) exciting light in the cortex and hippocampus. DAPI staining for nuclei (blue). White square showing the co-localization of an enlarged neuron (blue) with autofluorescence (green and red). **E** and **F** Quantification analysis of lipofuscin foci in the cortex (*p* < 0.0001, < 0.0001, < 0.0001) and hippocampus (*p* < 0.0001, < 0.0001, < 0.0001). The data are expressed as the mean ± SEM (*n* = 12 in each group). Two-way ANOVA followed by *Tukey’s *post hoc test or *Scheirer–Ray–Hare* test: **p < 0.01

Lipofuscin, also called “age-pigment”, is frequently found as aggregates in neurons of subjects with neurodegenerative disorders, caused by the accumulation of oxidized cross-linked proteins, lipids and sugars in the lysosomes of post-mitotic cells. Compared with age-matched control mice, there was a significant increase in autofluorescent lipofuscin deposits in the cortex and hippocampus of PIL mice at month 4, and these deposits further increased with time (Fig. 8). However, the density of neuronal nuclei (NeuN)-immunoreactive neurons in the cortex and hippocampus did not significantly differ between PIL and control mice (Additional file 1: Fig. S1). Furthermore, the terminal deoxynucleotidyl transferase dUTP nick end labeling (TUNEL) assay revealed no significant signs of apoptosis in either PIL or control mice (Additional file 2: Fig. S2).

## Discussion

To best represent human disease and investigate pathogenesis and novel treatment approaches, it is crucial to take full advantage of the available mouse models of spontaneous or inducible lupus-like disease. However, previous studies primarily focused on genetically susceptible animals to investigate spontaneous NPSLE [24]. Neuropsychiatric symptoms in the inducible wild-type models of lupus have been less studied. Here, we systematically evaluated whether PIL mice can be used as an environmental-related inducible model of NPSLE. Consistent with previous reports [18, 20, 21], PIL mice presented several peripheral lupus-like manifestations (Fig. 2). Notably, we identified some brain dysfunctions and neuropathological changes, as follows: (a) olfactory dysfunction and an anxiety- and depression-like phenotype; (b) cytokine and chemokine overproduction in the brain tissues; (c) BBB leakage; (d) IgG deposition in the choroid plexus and lateral ventricular wall, and co-localization of IgG with microglia; (e) changes in morphology and density of microglia and astrocytes in the hippocampus; and (f) lipofuscin deposition in the cortical and hippocampal neurons. Our results highlight the potential application of PIL mice for exploring the pathogenesis of environmental-related NPSLE.

In the present study, PIL mice lost their interest in biological odorants and showed decreased sensitivity to repellents (Fig. 3A). Spontaneous lupus-prone MRL/lpr mice display a constellation of behavioral deficits dependent on olfaction [25, 26]. Human lupus patients are also reported to have olfactory dysfunction [27] that is associated with disease activity and anti-ribsomal P protein antibody levels [28]. One interesting point of our results is that the olfactory dysfunctions appeared earlier than other behavioral deficits, suggesting that the olfactory neural system is highly sensitive to immune attack. This highlights the possibility that olfactory function tests might be an effective approach for early diagnosis of NPSLE. Similarly, olfactory dysfunction has been shown to be an early marker for neurodegenerative diseases, such as Parkinson’s disease [29], Alzheimer’s disease [30], or dementia with Lewy bodies [31], suggesting it may be helpful in the characterization of prodromal stages of disease and the prediction of clinical outcomes of neurodegenerative diseases. Additionally, affective deficits are frequent neuropsychiatric disturbances in NPSLE [32], and severely impact the patients’ quality of life. Similarly, numerous genetically predisposed mouse models of NPSLE also exhibit affective disorders. For example, the MRL/lpr strain presents depression-like behavior and deficits in cognitive function without anxiety-like behavior [8]. Furthermore, NZB/W F1 mice exhibit congenital abnormalities, anxiety-like behavior and decreased locomotor activity [12], and B6.Nba2 mice show a strong anxiety phenotype and a mild depression phenotype [33]. Recently, several neuropsychiatric manifestations have been reported in PIL mice, including learning and memory disturbance [17] and decreased spontaneous activities [18]. PIL mice in our current study also showed a decrease in total movement distance in the open field test and elevated zero maze test, indicating a reduction in spontaneous activity caused by musculoskeletal changes. Nonetheless, the significant decrease in the relative time spent in the center and open arms represents an anxiety-like phenotype in PIL mice from month 4 to 8 (Fig. 3B, C). Additionally, we observed a severe and consistent depression-like phenotype in PIL mice at month 4 to 8 (Fig. 3D). In line with previous findings [34, 35], our results confirm an association between olfactory dysfunction and depression-like behavior. However, no significant difference was found in the PPI, rotarod, novel object recognition or social novelty preference test (Fig. 4), indicating that sensory, motor coordination and cognitive functions are not severely impaired in PIL mice. Our results are consistent with clinical data showing that obvious olfactory dysfunction and mood disorders mostly occur in NPSLE patients, while sensory, motor and cognitive dysfunctions are infrequent or subtle [36]. Compared with genetic mouse models, such as MRL/lpr mice with the MRL background, which itself may be involved in the severity of neurobehavioral abnormalities [24], our PIL mice, without a specific genetic background, better mimic the clinical manifestations and are therefore more suited for investigating the neuroimmune mechanisms of NPSLE.

Several observations have suggested that cytokine dysregulation may contribute to depression-like behavior in NPSLE. First, increased levels of cytokines, especially IL-6, IL-8, IL-1, TNF-α and IFN-α, are found in the cerebrospinal fluid of NPSLE patients [37–40]. Second, the early dysregulation of cytokine production concords with the onset of depressive-like behavior in MRL/lpr mice [41] and other rodent strains [42, 43]. Third, anhedonia and other behavioral indices of depressive-like behavior in mice can be replicated by exogenous cytokines, such as IL-6 and TNF-α [41, 44], and are prevented by knockout of their receptors [44, 45]. In this study, we found substantial increases in cytokine and chemokine expression in the brain of PIL mice as early as week 2 (Fig. 5A, B). Because cytokine overexpression occurred earlier than behavioral deficits, it may be an initiating factor in the depressive-like behavior in PIL mice. It has been reported that neurons and glial cells in the brain are capable of secreting cytokines, such as IFN-γ, IL-1, IL-6, TNF-α and IL-10, in both their normal and pathological states [46]. Cytokine production in the brain can be promoted by various cytokines and chemokines, cytotoxic agents and neuronal debris, which can trigger a cascade of inflammatory processes. Overexpression of these cytokines has been suggested to be an important factor in the pathogenesis of neurotoxic and neurodegenerative disorders [47]. Pristane, known as a membrane-activating compound, can induce apoptosis in peripheral tissues and produce sufficient autoantigen substrates for immune intolerance, which can lead to overproduction of cytokines and the development of lupus-like autoimmunity. Cytokines, such as IL-1β and TNF-α, in the serum of PIL mice are capable of stimulating microglia to produce inflammatory cytokines and further drive the inflammatory cascade [48]. The increased peripheral inflammatory cytokines can disrupt the integrity of the BBB [49] and enter into the brain [50, 51]. In this study, we detected significant extravasation of Evans blue dye in the brain tissues, suggesting BBB leakage in PIL mice (Fig. 5C). Moreover, cytokines produced in the brain can also enter the serum across the damaged BBB. Thus, peripheral and central cytokines appeared to be integrated in the brain of PIL mice with a mutual promotion effect, which is difficult to be separated. Furthermore, we found IgG deposition in the choroid plexus and lateral ventricular wall (Fig. 6). A significant increase in IgG deposition has been reported in the hippocampus of PIL mice [19]. IgG deposition in the brain may occur following the impairment of the blood-ventricular barrier and choroid plexus-vascular barrier caused by cytokines in PIL mice. IgG deposition can promote endothelial damage, microglial activation and inflammatory mediator production, resulting in a positive feedback loop that disrupts immune homeostasis [52, 53]. Furthermore, we found a sign of phagocytosis of IgG by microglia (co-localization of IgG with microglia), suggesting that microglia may play a neuroprotective role in PIL mice.

Microglia are innate immune cells in the brain, connecting the nervous system with the immune system. Several studies have suggested that microglia are involved in the development of neuropsychiatric symptoms in NPSLE. First, microglia can be activated by cytokines, such as TNF-α, and inhibited by downregulating markers of microglial activation in MRL/lpr mice [48]. Second, microglia may initially migrate to the BBB to protect its integrity, and then transform into a reactive phenotype that damages the BBB and triggers neuroinflammation in NPSLE model mice [54]. Third, antagonism of microglial activation significantly attenuates spatial memory deficits and depression-like behavior in MRL/lpr mice [55]. In line with these observations, we found microglial activation during the initial stage of the disease in PIL mice, indicated by an increase in microglial density and specific morphological changes (Fig. 7B, D). It has been suggested that activated microglia may play a neuroprotective role during the early stage of neurodegenerative disorders [56]. Microglia can respond to the stimulation of neurotransmitters, such as adenosine triphosphate, glutamate and histamine, released by damaged neurons, and consequently increase phagocytic activity to clear unwanted debris, protein aggregates and soluble antigens, thereby reducing their damage to neurons [57]. Notably, we also found that microglial density declined sharply in the hippocampus of PIL mice from month 4 to 8. The sustained immune attack during the protracted progression of SLE may cause activation-mediated apoptosis of microglia [58]. The loss of microglia may severely hamper their ability to combat immune challenge and repair tissues, resulting in neural damage and behavioral deficits. Thus, loss of microglia over time following activation may play a critical role in the neuropathological changes in PIL mice. A recent study showed that neuronal degeneration or remodeling induced by the interaction between activated microglia and neurons may contribute to cognitive dysfunction [59]. A limitation of our study is the lack of detecting the markers of neuronal degeneration, such as neuronal complexity, length of dendrites and the number of spines in the brain. This shortcoming should be addressed in future studies. Together with our previous results, our findings suggest that dynamic change in microglia play an important role in the neuropathology of NPSLE, and therefore, modulating microglia may be a promising therapeutic strategy for NPSLE. In contrast, astrocytes were persistently activated throughout the observed course of the disease in PIL mice (Fig. 7C, E). Astrocytes generally interact with both neural and non-neural cells and perform dynamic activities crucial for neural circuit function, neurological function and behavior [60]. In neural disorders, astrocytes can be activated and produce nerve growth factors and immune mediators, such as IL-1 and nitric oxide, in response to brain inflammation or injury [61]. Histopathological investigations of NPSLE patient’ brains confirm the widespread presence of activated astrocytes along with microglia within the heterogeneous pathological tissue changes [62]. Astrocytes are an important component of the BBB, and activated astrocytes are a typical hallmark of BBB dysfunction [63]. In addition to the physiological role of astrocytes in synaptic refinement, activated astrocytes have also been implicated in pathological synapse loss and dysfunction following injury or nervous system degeneration in adults [64]. Overall, astrocyte activation may contribute to the neural dysfunction in PIL mice; however, further study is needed to clarify the underlying mechanisms.

Another important finding of our study is that lipofuscin deposits were found in neurons in both the cortex and hippocampus in PIL mice (Fig. 8). Lipofuscin, known as age pigments, are autofluorescent lipopigments formed by lipids, metals and misfolded proteins, which are especially abundant in neural cells. Lipofuscin within the brain increase not only with age, but also with pathological processes, such as neuronal dysfunction and a repertoire of cellular alterations, including oxidative stress, and proteasomal, lysosomal and mitochondrial dysfunctions [65–69]. Recent evidence suggests that lipofuscin may participate in the pathogenesis of various neurodegenerative disorders [70]. Furthermore, senescent neural cells accumulate in the hippocampus of the MRL/lpr SLE model mice with depressive behavior [71]. The increase in lipofuscin deposition in PIL mice may be attributable to the overexpression of cytokines in the brain, which can induce the synthesis of oxidative stress products. Accumulated oxidative stress products within neurons may orchestrate inflammation, disrupt the metabolism of lipids and metals, and thereby promote the generation of lipofuscin. Loss of neuroprotective effects from microglia may accelerate this pathological process during the progressive stage of SLE. Thus, lipofuscin deposition in neurons may be an important factor involved in neuronal damage in PIL mice. Though we observed neither a significant decrease in neuronal density by NeuN staining nor a sign of neural apoptosis by TUNEL staining of cortical and hippocampal slices, it is premature to exclude the possibility of neural damage considering the relatively low sensitivity of NeuN and TUNEL staining. The detailed mechanisms of neural damage need to be further explored in future studies.

**Fig. 9:**
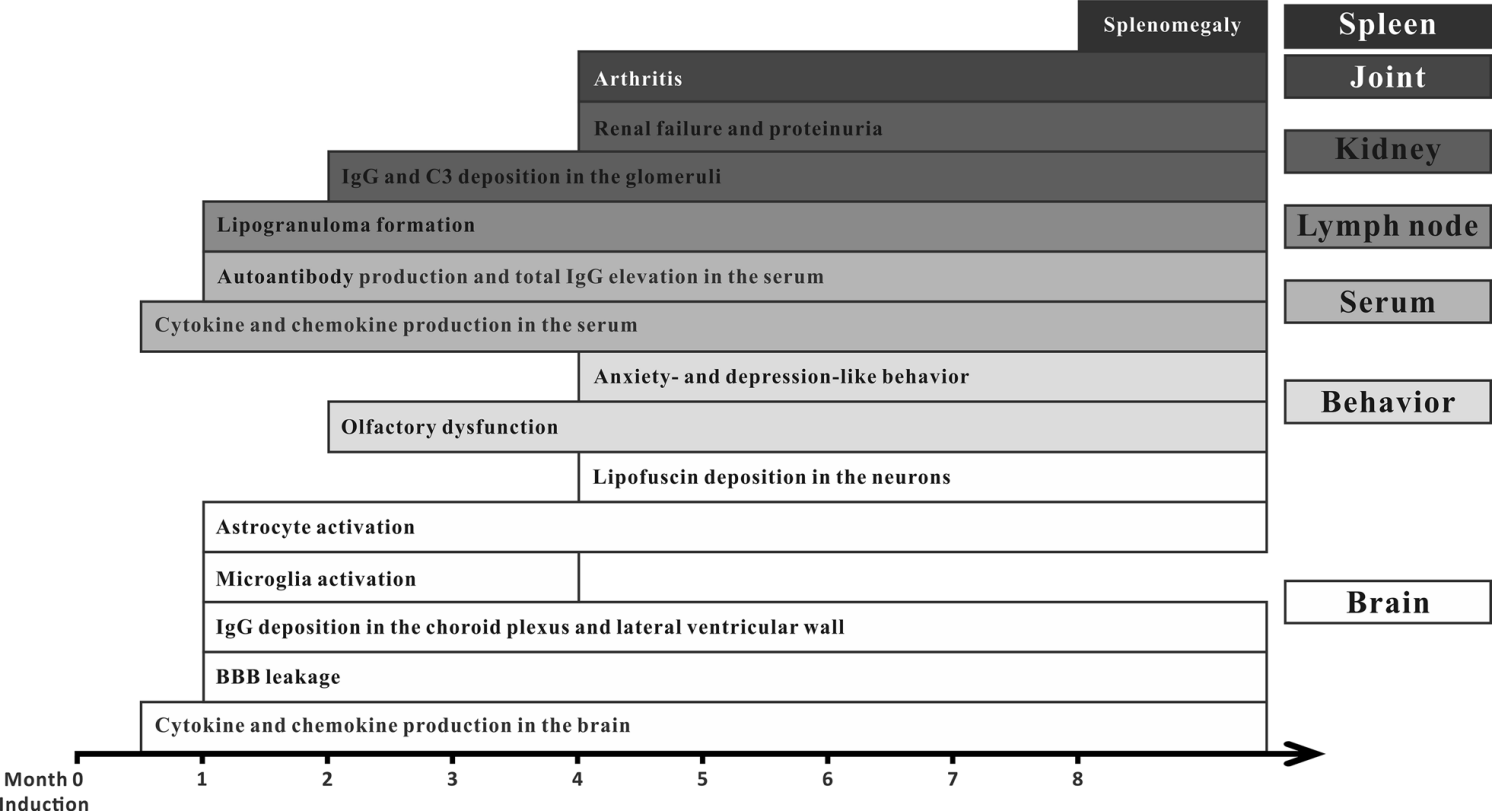
Peripheral manifestations, behavioral changes and brain pathogenic changes in PIL mice over the trial period

As shown in Fig. 9, the levels of cytokines and chemokines simultaneously increased in the brain and serum as early as week 2 after pristane injection. Initial histological changes in the brain, such as BBB leakage, IgG deposition and glial cell activation, appeared at month 1 after pristane injection, accompanied by peripheral signs of lipogranuloma formation, autoantibody production and total IgG elevation in the serum. Tissue damage commenced as olfactory dysfunction and IgG and C3 deposition in the glomeruli at month 2, and gradually progressed to anxiety- and depression-like behavior, renal failure and arthritis at month 4. Splenomegaly appeared at month 8. Therefore, the pathological changes in PIL mice may initiate from pristane-induced innate immune responses, which cause the overproduction of cytokines and chemokines, and enhance the production and release of autoantibodies. Cytokines in serum can disrupt the integrity of the BBB and facilitate the invasion of autoantibody IgGs into the brain, resulting in glial cell activation and neural dysfunction. Similar pathological mechanisms involving cytokines and IgGs also work in the glomerulonephritis and arthritis in PIL mice. Further study, using the transcriptomic profile comparisons between central and peripheral organs, such as brain, spleen, kidney and joint, may help elucidate the overlapping and distinct immune mechanisms in PIL mice and provide potential therapeutic targets for environmental-related SLE.

## Conclusions

We found that pristane can induce mice to exhibit olfactory dysfunction and an anxiety- and depression-like phenotype, along with increased expression of cytokines, BBB leakage, activation of microglia and astrocytes and aberrant deposition of IgG and lipofuscin in the brain. Our results suggest that brain dysfunction in PIL mice may initiate from the dysregulation of cytokines, which subsequently triggers BBB impairment, IgG deposition, glial activation and neuronal damage. These findings suggest that PIL mice are a promising model for NPSLE study, and highlight the important roles of glial cells in the pathogenesis of NPSLE. Glial cells, may therefore serve as a potential therapeutic target for NPSLE. Future studies should testify whether anti-inflammatory drugs such as retinoic acid [72] and coptisine [21], which have been shown to ameliorate renal and cardiovascular dysfunciton in PIL mice, can reverse the neuroinflammation and attenuate phenotypic changes in NPSLE.

## Methods

### Animals

Specific pathogen-free BALB/c mice were purchased from Vital River Laboratory (Beijing, China) at the age of 4 weeks. Female mice were used for the experiment at the age of 8 weeks. All animals were reared in standard animal cages under environmentally controlled laboratory conditions (12/12 h light/dark cycle, 22 ± 2 °C, 40–80% humidity) with ad libitum access to food and water. All efforts were made to minimize animal suffering. The animals were maintained and treated in compliance with the policies and procedures detailed in the “Guide for the Care and Use of Laboratory Animals” of the National Institutes of Health. The Animal Care and Use Committee of China Medical University reviewed and approved the animal experimental protocols and the treatment procedures (No. KT2018060).

### Pristane injection

As shown in Fig. 1, at the age of 8 weeks, mice were randomly divided into the following eight groups (*n* = 12 per group): 4 control groups (1 m, 2 m, 4 m and 8 m) and 4 PIL groups (1 m, 2 m, 4 m and 8 m), which received a single intraperitoneal injection of 0.5 ml phosphate buffer saline (PBS) or pristane (Sigma-Aldrich, St. Louis, MO, USA), respectively. The dose of pristane used in this study was based on a previous research [73]. Mice were sacrificed at month 1, 2, 4 or 8 after a battery of behavioral tests. Blood samples were obtained from the eyeball. Tissue samples of spleen, kidney, joint and brain were harvested for further examination. There was no early euthanasia of animals during the study.

### ELISA for brain cytokines and chemokines, and serum cytokine, total IgG and autoantibody detection

Mice were anesthetized and transcardially perfused with 0.1 M PBS (pH 7.5, 4 °C). Brains were harvested and dissected into the left and right hemispheres. One hemisphere was snap-frozen in liquid nitrogen and subsequently made into frozen sections for immunofluorescence staining, while the other hemisphere was homogenized on ice in PBS and centrifuged at 12,000 rpm for 15 min at 4 °C to remove cell debris. Supernatants were collected and stored at − 80 °C until assay. Serum was separated by centrifugation at 5000 rpm for 15 min at 4 °C. Cytokines and chemokines in brain tissues and serum were detected at week 1 or 2, or month 1, 2, 4 or 8 using IL-1β, IFN-α, IFN-β, IL-10, IFN-γ, IL-6, TNF-α, IL-17A, BAFF, CCL2 and CXCL10 ELISA kits (Boster & Biological Technology, Wuhan, China), TWEAK and APRIL DuoSet kits (R&D Systems, Minneapolis, MN, USA) and CCL7 ELISA kit (CUSABIO, Wuhan, China). Autoantibodies (anti-chromatin IgG (Inova Diagnostics, San Diego, CA, USA), anti-dsDNA IgG and anti-Sm IgG (CUSABIO), and anti-nRNP IgG (Alpha Diagnostics, San Antonio, TX)) and total IgG (Boster & Biological Technology) in serum were determined using commercially available kits per manufacturer’s guidelines. Each sample was tested at least three times, and the average value was taken.

### Renal function and 24 h proteinuria assessment

The levels of Scr and BUN were detected using Scr and BUN assay kits (Jiancheng, Nanjing, China). Experimental procedures were strictly followed according to the manufacturer’s protocols. Urine samples from mice in a metabolic cage were collected over 24 h. Proteinuria was measured with a BCA kit (Beyotime biotechnology, Shanghai, China). Each sample was tested at least three times and the average value was taken.

### Hematoxylin and eosin (H&E) staining

Tissues from the hind limb, brain and kidney were harvested and post-fixed in a solution of 4% paraformaldehyde in PBS overnight at 4 °C. The joint tissues were decalcified with ethylenediamine tetraacetic acid (EDTA, pH 8.0) for 2 weeks, and then embedded in paraffin after dehydration, and sliced into 4 μm sections. The sections were stained with H&E to observe synovial damage and provide an anatomic reference for the glomerulus and brain areas.

### Arthritis severity score and synovial inflammation score

For quantified scoring of arthritis severity, we used a previously published scoring system [16], as follows: score scale of 0–3, where 0 = normal, 1 = slight swelling or erythema of the wrist/ankle joint or footpad, 2 = moderate swelling and erythema of the wrist/ankle joint or footpad, and 3 = severe swelling and erythema of the paw. The scores for individual limbs were summed to obtain a total arthritis severity score of 12 per animal. Arthritis severity score was assessed twice by two independent observers. For synovial inflammation, we used a scoring system described previously [74], in which 5 high-power magnification fields (HPF) were scored for the percentage of infiltrative mononuclear inflammatory cells, as follows: 0 = absent, 1 = mild (1–10%), 2 = moderate (11–50%), and 3 = severe (51–100%). The average score of the 5 HPFs was used for analyses.

### Spleen index

Spleen index was calculated as the ratio of spleen weight to body weight (mg/g) as reported previously [18].

### Behavioral assessments

The mice were subjected to a series of behavioral tests in the following order: olfactory sensitivity test, open field test, elevated zero maze test, novel object test, social novelty preference test, rotarod test, PPI test and forced swim test. Mice were tested during the same lighting and time-of-day conditions. All behavioral chambers were cleaned with 70% ethanol as mice were changed. Researchers were blinded to the experimental groupings.

#### Olfactory sensitivity test

The paradigm used to assess olfactory sensitivity in this study was similar to that in an earlier report [26]. Following habituation to a new test chamber (45 cm × 24 cm × 20 cm), each mouse was introduced to a 5 cm × 5 cm piece of filter paper scented with 0.25 ml of an odorant for 2 min. Then, the scented filter paper was removed, and the mouse was allowed to rest for 1 min. This procedure was repeated three times. Each mouse was presented with repellents (vinegar and alcohol) and attractants (male feces and female feces) diluted with PBS to the same concentration. Test chamber was covered with a clear piece of plexiglas to limit evaporation and entry of external odorants. Active investigation was defined as directed sniffing within 0.5 cm of the odorant source and the sniffing time was recorded. Sniffing time for each trial was summed to obtain a total value per animal.

#### Open field test

The open field chamber (40 cm × 28 cm × 40 cm) was made up of black polyvinyl chloride panels with a non-reflective base. The central zone was defined as a 20 cm × 14 cm area. Each mouse was positioned individually in the center zone and allowed to freely explore the arena for 30 min. Total distance travelled (km) and time spent in the center (s) were digitally recorded and analyzed using custom-built programs.

#### Elevated zero maze test

The elevated zero maze was a ring-shaped apparatus, elevated 50 cm from the floor, and consisted of a circular platform (outer diameter 50 cm, width 10 cm) divided into four quadrants of equal length with two open arms and two closed arms (surrounded by a 20-cm wall from the surface of the maze). The test was conducted as previously described [75]. The test mouse was placed at the open arm and was allowed to conduct a 10-min free exploration. Total distance travelled (m) and percentage of time spent in the open arms were digitally recorded and analyzed by custom-built programs.

#### Novel object recognition test

The novel object recognition test was used to evaluate recognition memory and was conducted as previously described [76, 77]. During the acclimation phase, mice were allowed to habituate to the apparatus (55 cm × 40 cm × 30 cm) with no objects for 10 min, and then a test phase began 24 h later. On the trial day, two identical cylindrical objects were placed in the opposite side of the apparatus, and mice were allowed to spend 10 min with the objects. One hour later, one of the objects was replaced with a triangular object, and time spent exploring the novel and familiar object were digitally recorded for 10 min. The time spent in close interaction with each object was converted into a discrimination ratio, which was calculated as follows: time spent exploring the novel object/total time spent exploring both objects.

#### Social novelty preference test

The social novelty preference test was performed as previously described, with minor modification [78, 79]**.** During the acclimation phase, a test mouse was allowed to habituate to the apparatus for 10 min. A stranger mouse was then placed in one of the wire cages. The test mouse was allowed to spend 10 min to explore the entire apparatus. Subsequently, a novel stranger mouse was placed in the other wire cage. The test mouse was allowed to freely investigate the entire apparatus (the familiar mouse in one corner and the novel stranger mouse in the opposite corner) for 10 min. The time spent in close interaction with each mouse was digitally recorded and converted into a discrimination ratio, which was calculated as follows: time spent exploring the stranger mouse/total time spent exploring both mice.

#### Rotarod test

The rotarod test was used to evaluate motor coordination and was performed as previously described, with minor modification [18]. First, mice were placed on the stationary bar to habituate to the apparatus for 2 min. Then, the rotarod began to accelerate from 4 to 40 rpm. Latency to fall off the rotating rod was recorded with a 5-min cutoff time for three trials per day over 3 consecutive days and the mean retention time on the rod per trial was recorded.

#### PPI test

PPI test was measured using a startle chamber and was conducted as previously described, with minor modification [80, 81]. The test mouse was given a 10-min acclimation period in the startle chamber during which a 70 dB background noise was presented, and then the test mouse was subjected to test trials consisting of four trial types; that is, one type of startle stimulus only trial and three types of PPI trials. White noise of 120 dB (40 ms) was used as the startle stimulus for all trial types. The peak startle amplitude was recorded with the onset of the startle sound. The prepulse stimulus was presented 100 ms before the onset of the startle stimulus with an intensity of 75, 85 or 95 dB (20 ms). Six blocks of the four trial types were presented in a pseudorandom order such that each trial type was presented once within a block. The intertrial interval had an average duration of 15 s. PPI responses were calculated as follows: PPI% = [1 − (prepulse trials/startle only trials)] × 100.

#### Forced swim test

Each mouse was placed into a glass beaker containing 3000 ml of water maintained at approximately 24 ± 1 °C. Following habituation to swimming in this glass beaker for 2 min, a 4-min test session was digitally recorded. Mice placed in this situation had no way to escape, and began struggling and swimming, and eventually exhibited behavioral despair, assessed as immobility [34]. Depression-like behavior was defined assessed as the time spent immobile.

### Measurement of BBB permeability

To evaluate alterations in BBB permeability, Evans blue dye was used as a marker of BBB leakage, as previously described [82]. Briefly, mice were administered 2% Evans blue dye solution (4 ml/kg, Beyotime biotechnology) intravenously 30 min before sacrifice. Then, the brain tissues were homogenized in 50% trichloroacetic acid at a 1:3 v/v ratio. BBB permeability was assessed as Evans blue extravasation, and was quantified in the supernatant from each sample following addition of 90 μl of 95% ethanol (absorbance, 620 nm).

#### Immunofluorescence staining

Kidney and brain tissues were dissected, fixed in 4% paraformaldehyde for 24 h, followed by immersion in 30% sucrose (w/v) solution at 4 °C overnight. Tissues were then cut into 10-μm-thick coronal frozen sections, and blocked with 10% goat serum for 30 min at room temperature. Sections were incubated with primary antibodies, including anti-C3 (1:100; Santa Cruz, CA, USA), anti-Iba-1 (1:200; Abcam, Cambridge, UK), anti-GFAP (1:500; Abcam) or anti-NeuN (1:500; Abcam) at 4 °C overnight. On the following day, sections were incubated with secondary antibodies, including Alexa Fluor 488-conjugated goat anti-mouse IgG (1:200; Proteintech, Wuhan, China) or Alexa Fluor 488-conjugated goat anti-rabbit IgG (1:200, Proteintech, Wuhan, CHN) for 2 h at room temperature in the dark. For locating cell nuclei, sections were stained with DAPI (Beyotime biotechnology) for an additional 8 min. TUNEL staining was performed using a TUNEL Bright Green Apoptosis Detection kit (Vazyme, Nanjing, China) according to the manufacturer’s instructions. Images were captured on a microscope (BX53, Olympus, JPN) at × 200 or × 400 magnification. The number of stained cells was automatically counted in a defined area using Image J software. The MFI of C3 deposition in the glomeruli was calculated using Image J software. The data from three random sections for each individual mouse were averaged to obtain a single value. For IgG staining, sections were incubated with Alexa Fluor 594-conjugated goat anti-mouse IgG (1:200, Proteintech) for 2 h at room temperature in the dark. The MFI of IgG deposition in the choroid plexus, lateral ventricular wall or glomeruli was calculated using Image J software. To examine autofluorescent lipofuscin, regions of interest were captured at 480 nm and 550 nm exciting light. The mean gray value of autofluorescent lipofuscin was quantified with image J software.

#### Statistical analysis

GraphPad Prism V8 software (La Jolla, CA, USA) and SPSS 22.0 software (SPSS Inc., Chicago, USA) were used for statistical analysis. Before applying parametric statistics, all data were checked for the assumptions of normality using the D’Agostino-Pearson omnibus normality test. All data were expressed as the mean ± SEM. Differences in normally distributed data were detected by repeated measures analysis of variance (ANOVA) (with time as within factor and group [PBS, pristane] as between factor) followed by *Tukey's *post hoc test for multiple comparison. For non-parametric data, the *Scheirer–Ray–Hare* extension of the Kruskal–Wallis test [83] was used as a non-parametric equivalent of the two-way ANOVA. *p* < 0.05 was considered a statistically significant difference in all sampled groups.

## Supplementary Information

**Additional file 1: Figure S1.** Examination for neuronal density in the cortex and hippocampus. (A) and (C) Representative images of the cortex and hippocampus stained with H&E. (B) and (D) Representative images showing NeuN-immunoreactive cells in the cortex and hippocampus and quantitative analysis of neuronal density in the cortex (*p* = 0.5857, 0.1302, 0.7324) and hippocampus (*p* = 0.0650, 0.1922, 0.7277). The data are expressed as the mean ± SEM (*n* = 12 in each group). Two-way ANOVA followed by *Tukey’s *post hoc test or *Scheirer–Ray–Hare* test.

**Additional file 2: Figure S2.** TUNEL staining in the cortex and hippocampus. (A) and (C) Representative images of the cortex and hippocampus stained with H&E. (B) and (D) Representative images showing no TUNEL-immunoreactive cells in the cortex and hippocampus. DAPI staining for nuclei (blue).

## Data Availability

The data are available for any scientific use with kind permission.

## Abbreviations

SLE: Systemic lupus erythematosus
NPSLE: Neuropsychiatric lupus
BBB: Blood–brain barrier
PIL: Pristane induced lupus
IL: Interleukin
IFN: Interferon
TNF: Tumor necrosis factors-α
TWEAK: Tumor necrosis factor-like weak inducer of apoptosis
BAFF: B cell activating factor
APRIL: A proliferation inducing ligand
CCL: C–C motif chemokine
CXCL: C-X-C motif chemokine 10
Anti-dsDNA: Anti-double stranded DNA
Anti-nRNP: Anti-nuclear ribonucleoprotein
IgG: Immunoglobin G
C3: Complement 3
Scr: Serum creatinine
BUN: Blood urea nitrogen
MFI: Mean fluorescent intensity
PPI: Prepulse inhibition
Iba-1: Ionized calcium binding adapter molecule 1
GFAP: Glial fibriliary acid protein
Neun: Neuronal nuclei
TUNEL: Terminal deoxynucleotidyl transferase dUTP nick end labeling
H&E: Hematoxylin and eosin
EDTA: Ethylenediamine tetraacetic acid
HPF: High-power magnification fields
